# Allogeneic dendritic cells stimulated with antibodies against HLA class II polarize naive T cells in a follicular helper phenotype

**DOI:** 10.1038/s41598-018-22391-w

**Published:** 2018-03-05

**Authors:** Olivier Désy, Stéphanie Béland, Patrice Vallin, Julie Riopel, Eva Latulippe, Eric Wagner, Sacha A. De Serres

**Affiliations:** 1 Transplantation Unit, Renal Division, Department of Medicine, University Health Center of Quebec, Laval University, Québec, QC Canada; 2 Department of Pathology, University Health Center of Quebec, Laval University, Québec, QC Canada; 3 Immunology and Histocompatibility Laboratory, University Health Center of Quebec, Laval University, Québec, QC Canada

## Abstract

Follicular helper T cells (Tfh) are crucial for the production of high-affinity antibodies, such as alloantibodies, by providing the signals for B-cell proliferation and differentiation. Here, we demonstrate that human allogeneic dendritic cells (DC) stimulated with antibodies against HLA class II antigens preferentially differentiate human naive CD4^+^ T cells into Tfh cells. Following coculture with DCs treated with these antibodies, CD4^+^ T cells expressed CXCR5, ICOS, IL-21, Bcl-6 and phosphorylated STAT3. Blockade of IL-21 abrogated Bcl-6, while addition of the IL-12p40 subunit to the coculture increased CXCR5, Bcl-6, phosphorylated STAT3 and ICOS, indicating that they were both involved in Tfh polarization. We further phenotyped the peripheral T cells in a cohort of 55 kidney transplant recipients. Patients with anti-HLA-II donor-specific antibodies (DSA) presented higher blood counts of circulating Tfh cells than those with anti-HLA-I DSAs. Moreover, there was a predominance of lymphoid aggregates containing Tfh cells in biopsies from patients with antibody-mediated rejection and anti-HLA-II DSAs. Collectively, these data suggest that alloantibodies against HLA class II specifically promote the differentiation of naive T cells to Tfh cells following contact with DCs, a process that might appear *in situ* in human allografts and constitutes a therapeutic target.

## Introduction

Although the premature graft loss can be due to various causes, including infection, nephrotoxicity or recurrence of the primary renal disease^1,2^, alloimmunity remains the most common mechanism^2,3^. A report based on sensitive methods for detecting circulating anti-HLA antibodies suggested that up to 64% of graft losses could be due to rejection, mostly in the form of antibody-mediated rejection (ABMR)^3^. The most important physiopathologic component of ABMR is the presence of donor-specific antibodies (DSA), which often develop *de novo* following transplantation. Alloantibodies against HLA class II antigens are associated with high levels of endothelial-associated transcripts following tissue injury, and ABMR is mostly associated with this class of alloantibodies^4^. We and others have reported that antibodies against HLA class II are not only more commonly associated with chronic ABMR than antibodies against HLA class I, but are also predictive of graft loss^5–8^. Thus far, the reason that antibodies against HLA class II are associated with negative graft outcomes has not been elucidated.

B cells are responsible for producing anti-HLA antibodies; however, they need the help of T follicular helper lymphocytes (Tfh) to achieve this role^9^. In 2000, Tfh cells were first described as CD4^+^ T cells in human tonsils that express the chemokine receptor CXCR5^10–12^. In the lymph node, Tfh cells support B cell proliferation and provide signals that are crucial for the generation of high-affinity antibodies against specific antigens^12^. Tfh cells are notably characterized by the expression of the cell surface markers CXCR5 and ICOS, the cytokine IL-21 and the transcription factors Bcl-6 and STAT3^12,13^. In addition to playing a role in certain autoimmune diseases, such as systemic lupus erythematosus^14^ and juvenile dermatomyositis^15^, emerging data suggest a role for Tfh cells in mediating allograft rejection^16,17^.

In a recent publication, we studied the dendritic cells (DCs) infiltrating human kidney allografts^18^. In biopsies with a high DC density, immunofluorescence and electron microscopy studies showed direct physical contact between DCs and T cells, and the DC density correlated with higher Ki-67-positive labeling indices in infiltrating T cells. These observations suggest that the crosstalk between DCs and T cells may be driving an inflammatory response within the graft. Allograft transplantation is a human model of exposure to a persistent, large load of alloantigens from the donor. However, the interaction between DCs and T cells in this context remains poorly understood.

Based on these observations, we hypothesized that one of the mechanisms by which antibodies against HLA class II lead to increased graft loss is by preferentially instructing naive T cells to differentiate into Tfh cells through their interaction with DCs. We show, in a human allogeneic *in vitro* model, that HLA class II-stimulated DCs polarize naive CD4^+^ T cells into a Tfh phenotype. We further demonstrate in a cohort of kidney transplant recipients that patients with DSAs against HLA class II have higher frequencies of circulating Tfh cells and a higher number of lymphoid aggregates containing Tfh cells in their allograft biopsies than those with antibodies against HLA class I.

## Results

### Antibodies against HLA class II stimulate monocyte-derived DCs to mature into a CD80^+^CD86^hi^HLA-DR^+^BAFF^+^CCR7^+^ phenotype

**Figure 1:**
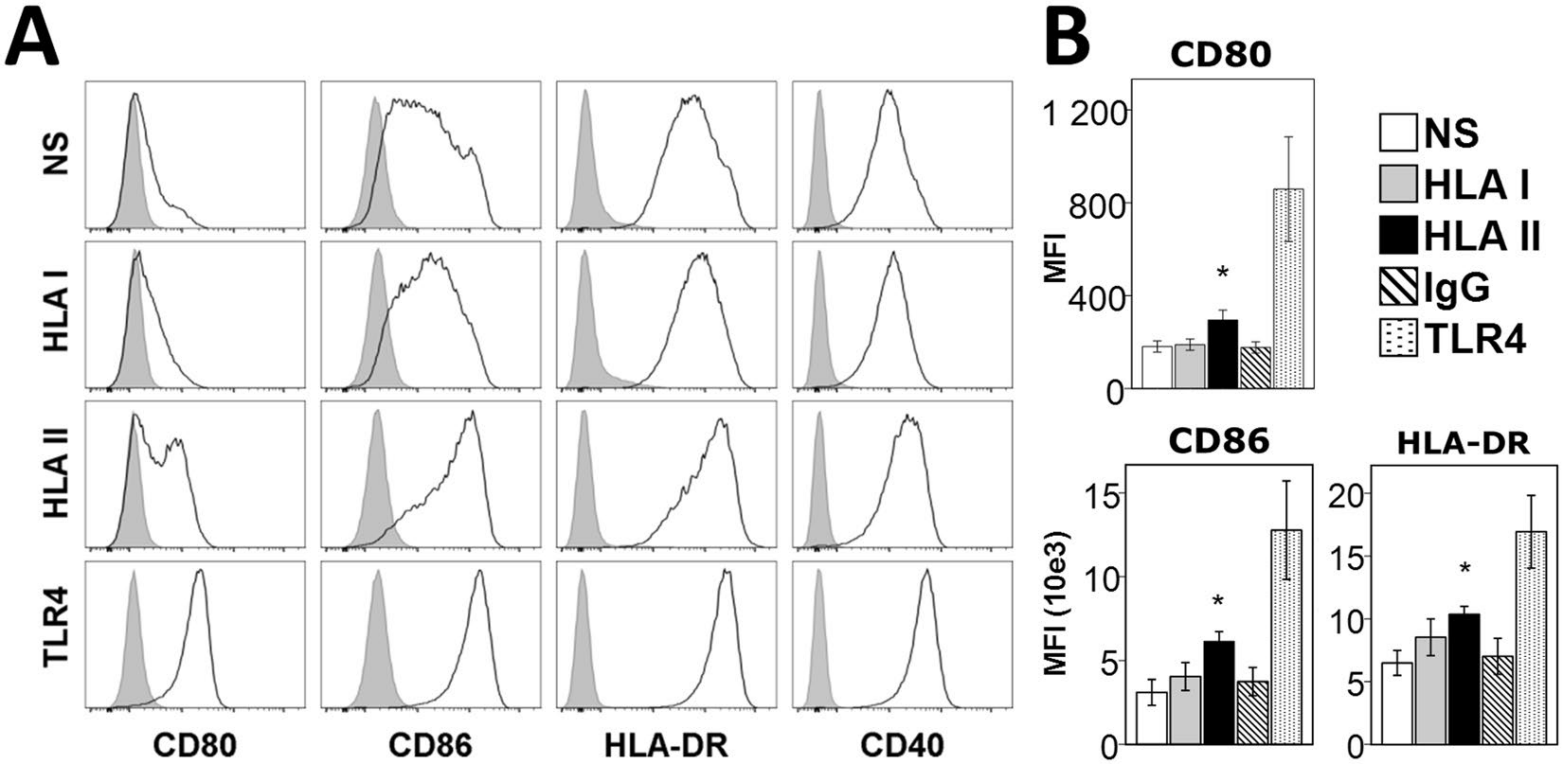
Phenotypes of moDCs after stimulation with HLA I and HLA II antibodies. (**A**) Flow cytometry analysis of the surface phenotype of moDCs following stimulation for 48 h. Closed gray histograms indicate isotype control and black line histograms indicate markers of interest. One representative experiment out of 11 performed is shown. (**B**) Summarized data of Median Fluorescence Intensity (MFI) for CD80, CD86 and HLA-DR in differentiated moDCs. The means ± SEM of 11 experiments are shown. *p < 0.05.

To investigate the effect of HLA I and HLA II on the DC phenotype, CD14^+^ monocytes from healthy volunteers were isolated and differentiated into immature DCs using GM-CSF and IL-4. The cells were then matured under the following conditions: unstimulated, stimulated with a pan-antibody against HLA class I, a pan-antibody against HLA class II, a corresponding IgG2a isotype or TLR4 (LPS). Generation of monocyte-derived DCs (moDCs) was confirmed by CD11c expression (≥95% of cells). The differentiation of moDCs into mature DCs with an APC phenotype predominantly occurred in the presence of antibodies against HLA class II (HLA-II-moDCs), as shown by the higher levels of CD80, CD86 and HLA-DR mean fluorescence intensity compared to unstimulated cells (Fig. 1A,B).

### HLA-II-moDCs polarize CD4^+^ naive T cells in CXCR5^+^IL21^+^ cells

**Figure 2:**
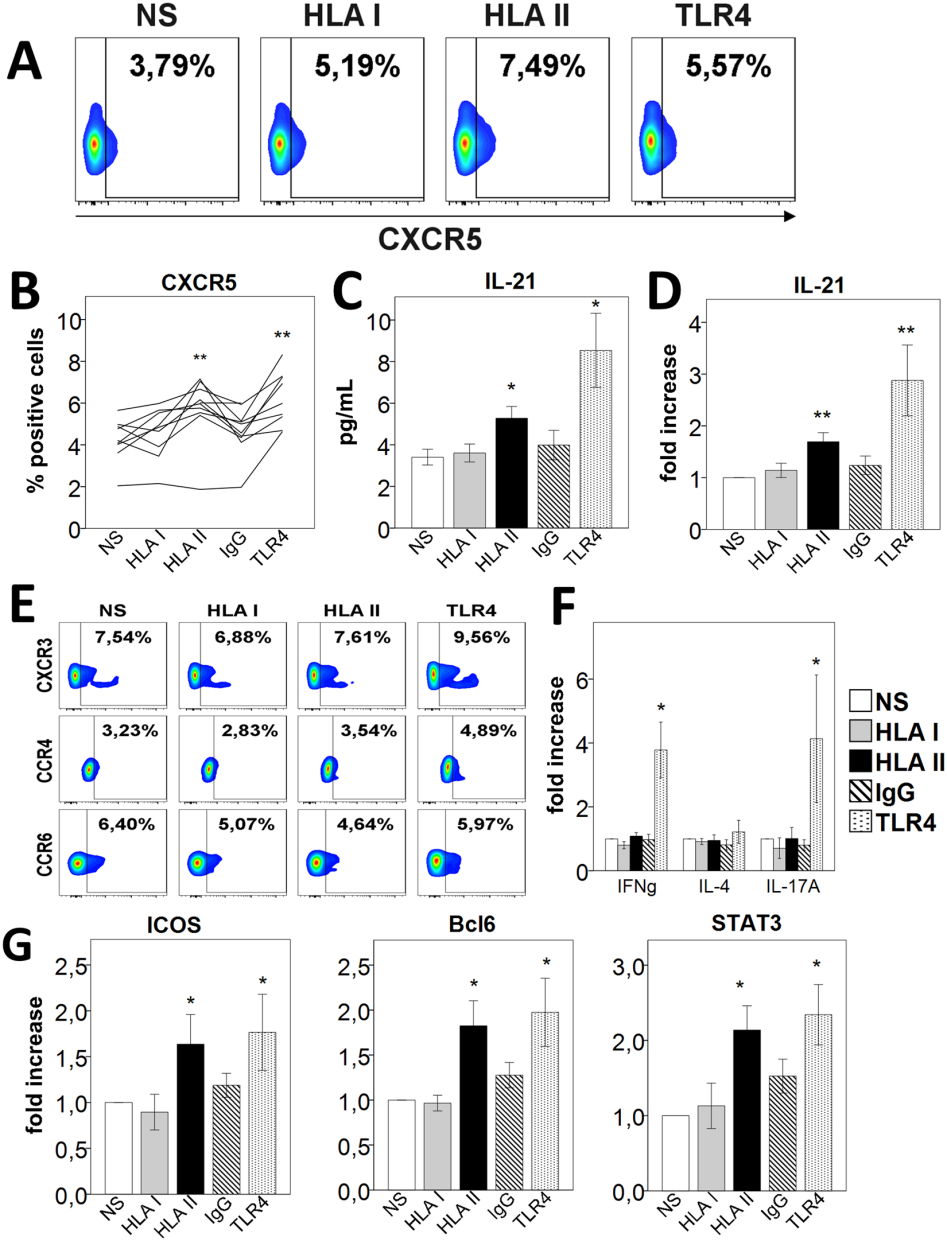
HLA-II-moDCs polarize CD4^+^ naive T cells into a Tfh phenotype. In all experiments, CD4^+^ naive T cells were cocultured with stimulated moDCs for 6 days. (**A**) Flow cytometry analysis of CXCR5 surface expression (gated on CD4^+^cells). One representative experiment out of 9 performed is shown. (**B**) Summary analysis of the CXCR5 percentage expression. Each line represents a single experiment (n = 9). (**C**) Cell culture supernatants were analyzed for IL-21 production by ELISA (mean ± SEM, n = 9). (**D**) Summarized data for intracellular IL-21 levels by flow cytometry. Values are expressed as the fold increase in IL-21 levels compared with levels in untreated cells (mean ± SEM, n = 13). (**E**) Flow cytometry analysis for Th1 (CXCR3), Th2 (CCR4) and Th17 (CCR6) surface marker expression (gated on CD4^+^ cells). One representative experiment of 9 was shown. (**F**) Coculture supernatants were analyzed for IFN-γ, IL-4 and IL-17A levels by ELISA. Values are expressed as fold increase in cytokine levels compared with levels in unstimulated cells (mean ± SEM, n = 9). (**G**) Flow cytometry analysis for the surface expression of ICOS and the intracellular expression of Bcl-6 and pSTAT3 in CD4^+^45RA^+^ T cells stimulated with moDCs for 6 days (mean ± SEM, n ≥ 4). *p < 0.05; NS, non-stimulated; IgG, isotype control.

We then sought to examine whether the HLA-II-differentiated moDCs polarize naive T cells into the Tfh phenotype. We performed allogeneic cocultures with moDCs using purified naive CD4^+^ T cells isolated from a healthy volunteer different that the one from whom monocytes were collected. We analyzed surface phenotype and cytokine production for the Tfh, Th1, Th2 and Th17 phenotypes after 6 days of coculture. HLA-II-moDCs induced a substantially increased expression of CXCR5 in CD4^+^ T cells (Fig. 2A,B). Concomitantly, we observed higher levels of IL-21 secreted in the coculture supernatants from HLA-II-moDCs compared with unstimulated-moDCs and HLA-I-moDCs (Fig. 2C), results confirmed by the analysis of the intracellular expression of the cytokine in CD4^+^ T cells (Fig. 2D) and CD4^+^CXCR5^+^ T cells (Supplementary Figure S1). We found no difference in the proportion of CD4^+^ T cells expressing CXCR3, CCR4 or CCR6 on their surface (Fig. 2E). There was also no significant difference in the secretion levels of IFN-γ, IL-4 and IL-17 in the cell culture supernatants from T-cells (Fig. 2F), indicating that the naive T cells were not polarized into a specific Th1, Th2 or Th17 phenotype.

We next looked whether the HLA-II-differentiated moDCs have an impact on cell proliferation. CFSE-labelled naive CD4^+^ T cells were stimulated with differentiated moDCs for 6 days before the analysis by flow cytometry. HLA-II-moDCs increased CD4^+^ T cells proliferation in comparison with the unstimulated-moDCs, as measured by CFSE intensity (Supplementary Figure S2). We then tested whether the polarization to the Tfh phenotype was consistent across various concentrations of antibodies against HLA class II during the differentiation and maturation of moDCs. We induced the maturation of DCs using antibody concentrations of 0.5, 1.0 and 2.0 µg/mL. Given that we used a pan-HLA antibody, these concentrations are likely to capture the physiological concentration found in the blood of transplant recipients with anti-HLA alloantibodies^19^. CXCR5 expression was present at the 0.5 µg/mL antibody concentration and had fairly similar levels at 1.0 and 2.0 µg/mL concentrations (data not shown). Collectively, these data indicate that antibodies against HLA-II-differentiated moDCs stimulate the proliferation of naive T cells and their differentiation into Tfh cells.

### Confirmation that HLA-II-moDCs polarize CD4^+^ naive T cells in a Tfh phenotype

To further confirm that naive CD4^+^ T cells cocultured with allogeneic HLA-II-moDCs polarized into the Tfh phenotype, we characterized the surface expression markers, cytokine profiles and transcription factors associated with this T cell lineage. We noted a higher proportion of ICOS-positive cells in the CD4^+^ T cells induced with HLA-II-moDCs (Fig. 2G). There was also a significant increase in Bcl-6 and phosphorylated STAT3 (pSTAT3) expression in this allogeneic coculture (Fig. 2G). We looked at the expression of Bcl-6 within the CD4^+^CXCR5^+^ population. Here also, the expression was higher in this population (Supplementary Figure S3).

Next, we carried out an extended experimental protocol in which T cells were submitted to a second round of stimulation with differentiated moDCs after the first 6 days of coculture. T cells were kept in this second allogeneic culture for an additional 6 days and then reexamined (not shown). We observed a similar surface phenotype in this protocol, confirming that T cells maintain their Tfh commitment. Altogether, these data indicate that the allogeneic coculture with HLA-II-moDCs leads to the differentiation of naive CD4^+^ T cells into Tfh cells.

### Regulation of the expression of Tfh-specific transcription factors and surface phenotype

**Figure 3:**
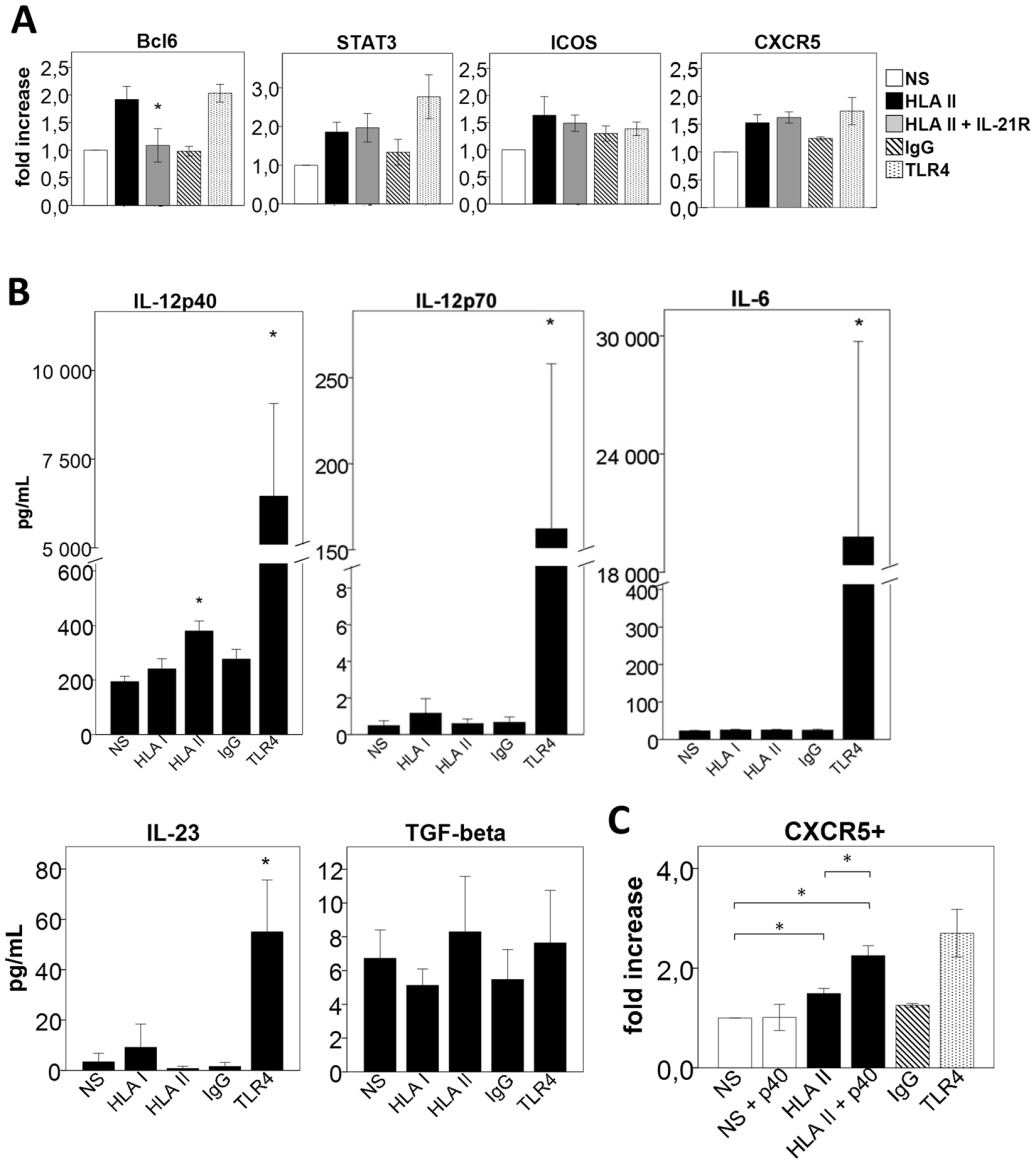
Regulation of the expression of Tfh-specific transcription factors and surface phenotype. (**A**) Flow cytometry analysis of intracellular Bcl-6, pSTAT3 and surface expression of ICOS and CXCR5 in CD4^+^ T cells stimulated with allogeneic unstimulated moDCs (NS), HLA-II-moDCs (HLA II), HLA II + IL21R-moDCs (HLA II + IL21 inh), IgG2a-moDCs (IgG) and TLR4 for 6 days. Values are expressed as fold increase in marker levels compared to levels in unstimulated cells (mean ± SEM, n = 4). The mean ± SEM of 4 experiments are shown. (**B**) Cytokine levels measured in supernatants of DC cultures (mean ± SEM, n = 10). (**C**) Flow cytometry analysis of CXCR5 in CD4^+^ T cells stimulated with NS, NS + p40 subunit of IL-12, HLA II, HLA II + p40 subunit of IL-12, IgG and TLR4 for 6 days (mean ± SEM, n = 5). *p < 0.05; NS, non-stimulated; IgG, isotype control.

In mice, it has been demonstrated that IL-21 is the predominant cytokine responsible for upregulating Bcl-6 expression, which then leads to Tfh differentiation and B cell maturation in germinal centers^20,21^. In humans, the importance of the IL-21 cytokine in the differentiation of Tfh cells is still unclear. It has been demonstrated that multiple cytokine pathways, including IL-21, are involved in the differentiation of human Tfh cells^22^. We thus tested the effect of IL-21 blockade on the expression of Bcl-6, pSTAT3, ICOS and CXCR5 in Tfh cells generated in HLA-II-moDCs allogeneic cultures. Adding the anti-IL-21 antibody in the coculture effectively abrogated Bcl-6 expression in CD4^+^ T cells (Fig. 3A). We observed a similar effect of IL-21 inhibition in CFSE-labelled CD4^+^ T cells (data not shown). However, the IL-21 blockade changed neither the expression of pSTAT3 nor the surface expression of ICOS or CXCR5 (Fig. 3A). In all, these data indicate that IL-21 is involved in the upregulation of Bcl-6 expression in humans.

The absence of effect on pSTAT3 expression observed following blockade of IL-21 suggest that, in humans, other cytokines may be critical for the differentiation of CD4^+^ cells into Tfh cells. For instance, it has been reported that IL-12, IL-6, IL-23, and TGF-β could contribute to the STAT3-mediated differentiation in Tfh cells^9,23^. We thus analyzed the expression of these cytokines in the supernatant of the differentiated moDCs. We noted a nearly two-fold increase in the expression of IL-12p40 (194 ± 19 vs. 381 ± 37, p < 0.05) in the supernatant of HLA-II-moDCs compared with the unstimulated control, but no difference in the expression of the other cytokines (Fig. 3B).

The p40 subunit signals through the IL-12Rβ1 chain found in the receptors of IL-12 and IL-23^24^. The importance of IL-12 and the receptor molecule IL-12Rβ1 have been highlighted in the generation and the function of Tfh cells in humans^25,26^. Based on the observation above in the supernatants of moDCs, we examined the effect of p40 in the DC-T cell coculture. The addition of this monomeric subunit to the anti-HLA-II treated moDC-T cell coculture resulted in a significant increase in the surface expression of CXCR5 (Fig. 3C). In addition, p40 enhanced the expression of Bcl-6, pSTAT3 and ICOS (Supplementary Figure S4). Collectively, these results indicate that the p40 subunit promotes the Tfh phenotype.

### Kidney transplant recipients with alloantibodies against HLA class II exhibit higher levels of peripheral Tfh cells

We next sought to characterize the frequency of circulating Tfh cells in kidney transplant recipients. We used samples from a prospective observational cohort of 55 kidney transplant recipients from whom PBMCs were collected concurrently with an allograft biopsy and donor-specific antibody detection on the same day. This cohort consists mostly of middle aged (mean 48, SD 14 years), male (51%) recipients of a first kidney transplant (85%). Blood sample collections and allograft biopsies were taken at a median time of 96 (25–75th percentiles 21–136) months post-transplant. Most patients were kept on a triple maintenance immunosuppressive regimen consisting of prednisone, a calcineurin inhibitor and mycophenolate. Patients were categorized first according to the biopsy results into the following groups: non-rejectors (n = 7), ABMR (n = 12), T-cell mediated rejectors (TCMR) or borderline changes suspicious for acute TCMR (n = 26, using the tubulitis score of 1 and the interstitial inflammation of 0 as the threshold), recurring glomerulonephritis (n = 5) and evidence of infection at biopsy (acute bacterial pyelonephritis or polyomavirus nephropathy, n = 5). Patients with ABMR were further classified based on the HLA class of their respective DSAs. Complete blood counts were obtained from the clinical lab on same the day as the PBMC collection to derive absolute CXCR5^+^ counts within the total CD4^+^, as well as central memory (CD4^+^CD45RO^+^CCR7^+^) and peripheral memory (CD4^+^CD45RO^+^CCR7^−^) compartments.

**Figure 4:**
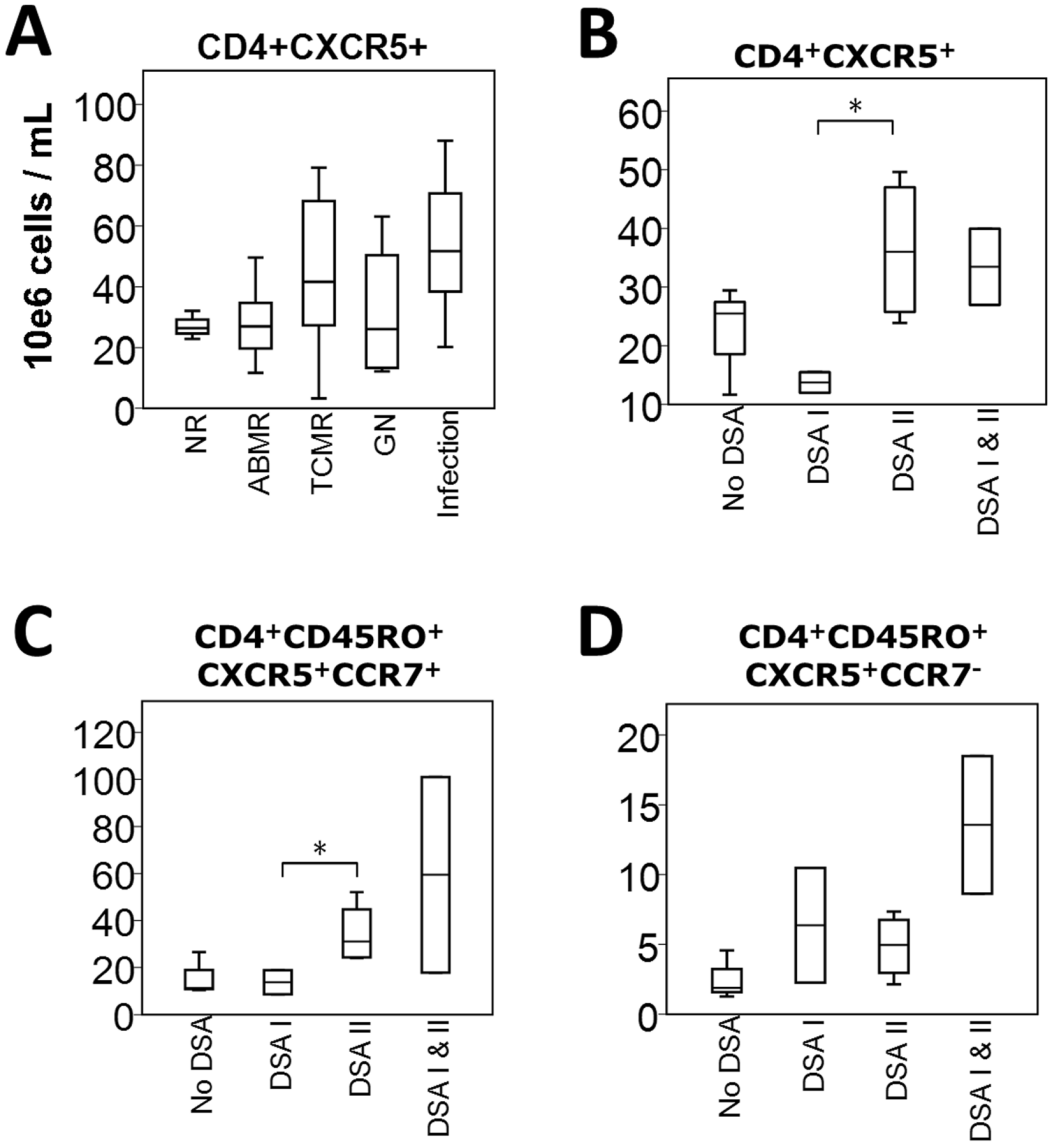
Relationship between absolute blood cell counts and the clinical status of kidney transplant recipients. (**A**) Boxplots (25th, median, 75th percentile) of CD4^+^CXCR5^+^ absolute blood counts are shown for non-rejectors (NR), antibody-mediated rejectors (ABMR), T-cell mediated rejectors (TCMR), patients with recurrent autoimmune glomerulonephritis (GN) and patients with infection (n = 55 total). (**B**–**D**) Comparison of absolute CD4^+^CXCR5^+^, CD4^+^CD45RO^+^CXCR5^+^CCR7^+^ and CD4^+^CD45RO^+^CXCR5^+^CCR7^-^ blood counts in patients with ABMR. We compared blood cell counts among patients with no circulating DSAs, patients with DSAs against HLA class I, patients with DSAs against HLA class II and patients with DSAs against both HLA classes (n = 12 total). *p < 0.05; DSAs, donor-specific antibody.

We first compared the frequency of circulating CD4^+^CXCR5^+^ T cells according to the histological diagnosis. We observed no significant difference between the groups compared (Fig. 4A). Similar results were obtained for central and peripheral memory analysis subsets (not depicted). Second, we focused on patients with ABMR. The CXCR5^+^ cell counts were higher in patients with DSAs against HLA class II than those of patients with DSAs against HLA class I within the total CD4^+^ and central memory T cell compartments (Fig. 4B,C) but were similar within the peripheral memory cells (Fig. 4D). Overall, these data suggest that patients with ABMR do not have increased frequencies of circulating Tfh. However, patients with DSAs against HLA class II have higher levels of circulating Tfh cells than those with DSAs against HLA class I, and these mostly belong to the central memory type.

### Kidney transplant recipients with DSAs against HLA class II exhibit higher levels of intragraft Tfh cells

**Figure 5:**
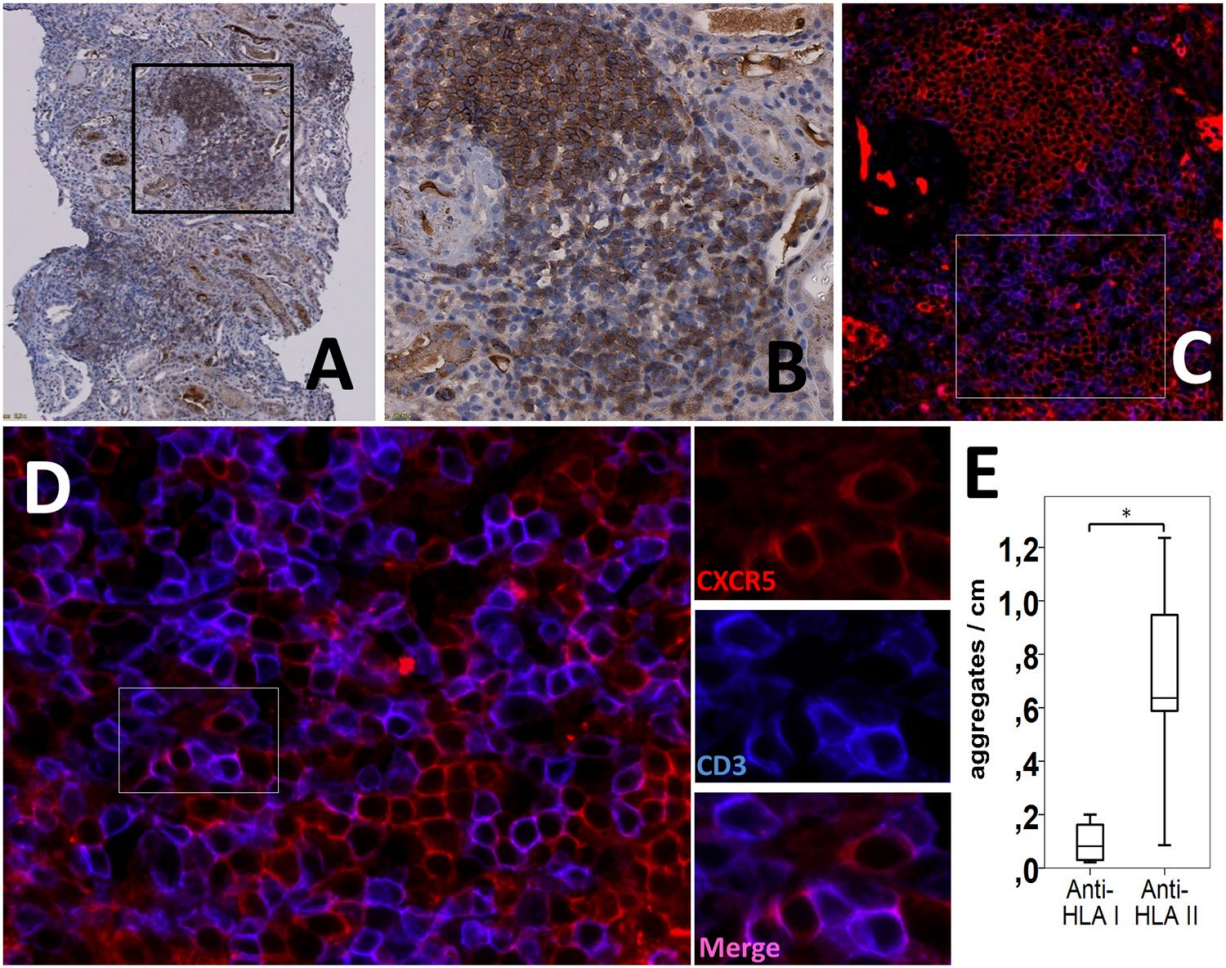
Tfh cells infiltrate the allograft of patients with circulating anti-HLA class II DSAs. (**A**,**B**) Representative photomicrographs (×10 and 20) of CXCR5^+^ cells (brown) in a patient with ABMR and circulating anti-HLA class II DSAs by immunohistochemical staining. (**C**,**D**) Dual immunofluorescence (× 20 to 80) staining of the same area as shown in B and high-power magnifications confirms the presence of Tfh cells by colocalization of CXCR5 (red) and CD3 (blue) in lymphoid aggregates. Some areas of the infiltrate were enriched with CD3^−^CXCR5^+^ B cells, whereas CD3^+^CXCR5^+^ T cells were scattered in other zones. (**E**) Summarized data (boxplots of 25th percentile, median and 75th percentile) for the number of aggregates containing CD3^+^CXCR5^+^ T cells per cm of tissue in patients with ABMR and antibodies against HLA class I (anti-HLA I) compared to antibodies against HLA class II (anti-HLA II) DSAs (n = 9). *p < 0.05.

To further assess the clinical relevance of these data, we examined whether Tfh cells were present in the allografts of patients with DSAs and whether there was a relationship between intragraft Tfh cells and the HLA class of alloantibodies. We used banked biopsies from kidney recipients with circulating DSAs against HLA class I or II. We first examined the number of CXCR5^+^ T cells in the allograft tissue by immunohistochemistry staining (Fig. 5A,B). Colocalization studies of CXCR5 and CD3 by immunofluorescence confirmed the presence of CD3^+^CXCR5^+^ Tfh cells and CD3^−^CXCR5^+^ B cells (Fig. 5C,D). In patients with DSAs against HLA class II, we found large numbers of lymphoid aggregates containing CD3^+^CXCR5^+^ T cells, but these were only rarely observed in patients with DSAs against HLA I (Fig. 5E). Staining of a native kidney nephrectomy showed no CXCR5^+^ cells in the tissue (not shown).

## Discussion

In this study, we examined the role of antibodies against HLA class I and class II in the differentiation of naive T cell into Tfh cells through their interaction with DCs. Our results show that antibodies against HLA class II are better than anti-HLA class I to mature DCs in an APC phenotype. Moreover, HLA class II-stimulated DCs preferentially polarize naive CD4^+^ T cells into Tfh cells. IL-21 blockade seems to partially abrogate the Tfh phenotype by decreasing the expression of the transcription factor Bcl-6. Examination of the cytokines produced following moDC differentiation showed that the p40 subunit of IL-12 was elevated when moDCs were treated with anti-HLA class II, and the addition of p40 monomer to the DC-T cell coculture promoted the expression of CXCR5, ICOS, Bcl-6 and pSTAT3. We further demonstrate that patients with circulating DSAs against HLA class II have higher levels of Tfh cells in the blood, mostly of the central memory type, and present Tfh cells inside lymphoid aggregates in their allograft. These data are clinically relevant because they suggest a mechanistic role for Tfh cells in ABMR, particularly in the presence of DSAs against HLA class II.

It is now clear that the presence of DSAs *de novo* post transplant is a major risk factor of poor outcomes^27^. Currently, ABMR, especially the chronic form, is an immunological point of no return: once DSAs are formed, the recipient is somewhat ‘vaccinated’ against his or her graft. Because the major barrier to improving long-term outcomes in solid organ transplantation remains chronic rejection, the cross-talk between DCs and naive T cells seems a novel therapeutic target to impede Tfh formation and prevent the generation of these alloantibodies. Our results show that, in humans, IL-21 and p40 blockade seems to be promising. Both targets have already been developed for other clinical indications^28,29^.

So far, little is known about the specific role of Tfh cells in ABMR. In humans, de Graav *et al*. showed that the numbers of circulating Tfh cells 3 months post-transplantation were higher in patients with pre-existing DSAs compared with those without^30^. Liarski *et al*. found at least 1 Tfh cell per high power field in 64% of biopsies with TCMR and 50% of biopsies with mixed rejection^31^. In a murine model of chronic graft-versus-host disease (GVHD), the frequency of Tfh cells correlated with the severity of GVHD^20^. In this model, blockade of IL-21 or of the IL-21 receptor prevented GVHD, while anti-B cell therapy was unsuccessful, suggesting that the process was Tfh-dependent whereas B-cell blockade was insufficient. Recently, de Leur *et al*. had nicely shown that stimulation of Tfh and memory B cells with donor alloantigen resulted in the formation of IgM and IgG producing plasmablasts^32^. In contrast to our design focusing on the APC-T cell interaction, de Leur’s model used sorted CD3^+^CD4^+^CXCR5^+^ and CD19^+^CD27^+^ from peripheral blood and used them in coculture. They found that the IL-21 blockade significantly inhibited the B cell differentiation and immunoglobulin secretion, but had not effect on the phenotype of Tfh cells.

The role and the specific function of the p40 monomer in transplantation remains poorly characterized. It is secreted in response to infectious and noninfectious inflammatory signals including disease states^33–35^. It has been shown to promote allograft rejection in a murine heart transplant model^36^. Interestingly, it inhibited the Th1 development in CD4^+^ T cells, but whether or not it influenced other CD4^+^ T cell phenotype or resulted in alloantibody production was not studied^36^. The p40 and p80 subunits have also been reported to be involved in innate immune cell migration^33^. Our results suggest a key role of p40 monomer in the formation of human Tfh.

The magnitude of IL-21 increase observed here is consistent with the fact that it stimulates bystander cells, Tfh cells secreting it in autocrine manner^12,37^. Besides IL-21, IL-6 and IL-10 are key regulators of STAT3 and it is known that production of IL-6 by DCs is associated with CD40 signaling^38,39^. It is possible that IL-6 produced not only by DCs, but following the DC-T cell interaction, initiates the polarization into Tfh cells. This would provide a likely explanation to the absence of effect of IL-21 blockade on pSTAT3 expression here. Additional studies are underway to elucidate the role of IL-6 in this model.

There are some limitations to this study. First, the percentages of CD4^+^ Tfh cells obtained in the model were low. However, this was expected from the experimental design which reproduces the ‘direct alloresponse’ occurring early, during the first years post transplant, when donors DCs are still be present in the recipient and interact directly with T cells. It is known that the frequency of peripheral T cells that react against a specific alloantigen varies mostly between 0.01 and 1%; in some animal models it can be higher than 1%^40^. Our results are within this range. This model is clinically relevant, because donor DCs may be found in the graft for up to 4 years post transplant^18^. We need to emphasize that, in contrast to other models in which the reaction was artificially enhanced by non-specific sitmulators^22,41^, we did not use stimulatory cytokines nor anti-CD3/CD28 beads. Second, the aim here was to characterize the generation of Tfh cells in an allogeneic model in the presence of DCs^18^. An extended, 3-week experimental protocol will be needed to assess the differentiation of B cells and the production of immunoglobulins beyond the Tfh commitment.

The findings reported in this article provide evidence of an association between antibodies against HLA class II and formation of Tfh cells following contact with allogeneic DCs, the level of circulating Tfh cells in the blood of kidney recipients and the numbers of Tfh cells infiltrating the allograft. These observations suggest that this process might appear *in situ* in human allografts. Additional studies will help to determine the precise impact of the HLA II subclass (DP, DQ, and DR) in Tfh formation, which may eventually lead to focused, personalized therapies based on Tfh blockade in clinical transplantation.

## Materials and Methods

### Monocytes and CD4^+^ T cells

We generated human Tfh cells using a 14-day coculture model (Supplementary Figure S5). PBMC samples were isolated from fresh heparinized blood of healthy volunteers by density gradient centrifugation using Ficoll-Paque PLUS (GE Healthcare, Uppsala, Sweden). Monocytes were purified (>95%) using the Pan Monocyte Isolation Kit (negative selection; Miltenyi Biotec Inc., Auburn, CA**)**. Total CD4^+^ T cells were purified (>95%) using the CD4^+^ T Cell Isolation Kit (negative selection; Miltenyi). Naive CD4^+^ T cells (CD45RA^+^) and memory CD4^+^ T cells (CD45RO^+^) were separated (>95%) using the CD45RA MicroBeads (positive selection; Miltenyi).

### Dendritic cells

Immature DCs were generated by culturing healthy monocytes in an X-vivo 15 free serum medium (Lonza, Walkersville, MD) supplemented with 1000 U/ml GM-CSF (Miltenyi) and 500 U/ml IL-4 (Miltenyi) for 6 days. The maturation of DCs was induced by adding to the medium one of the following stimulants: 100 ng/ml LPS (Sigma-Aldrich, St-Louis, MO), 0.5 μg/ml mouse IgG2a anti-human monoclonal HLA class I antibody clone W6/32 (Sigma-Aldrich), 0.5 μg/ml mouse IgG2a anti-human monoclonal HLA-DR, DP, DQ clone Tü39 (BD Biosciences, San Diego, CA), or 0.5 μg/ml mouse IgG2a isotype control (Sigma-Aldrich and BD Biosciences). The supernatants were collected at the end of the DC maturation stage for all samples.

### Coculture

1 × 10^6^ CD4^+^CD45RA^+^ or CD4^+^CD45RO^+^ T cells were cocultured with 1 × 10^5^ DCs in an X-vivo 15 free serum medium (Lonza). In some experiments, recombinant Human IL-21R Fc Chimera Protein 10 μg/ml (R&D Systems Inc., Minneapolis MN) and the p40 monomeric subunit of IL-12 300 pg/ml (Preprotech, Rocky Hill NJ) were added. On day 6 of culture, the supernatants were harvested, and CD4^+^ T cells were stimulated with anti-CD3/CD28 beads (Miltenyi) for 5 h. Then, CD4^+^ T cells were stained per the manufacturer’s instructions.

### Antibodies, reagents, and flow cytometry

For the DC phenotype, the following antibodies were used: anti-CD14-FITC, anti-CD11c-VioBlue, anti-CD80-PE, anti-CD86-PerCP-Vio700 (Miltenyi), anti-HLA-DR-APC-Cy7, anti-CCR2-APC, anti-CD16-Brilliant Violet^TM^ 510, anti-CCR7-FITC, anti-CCR5-PerCP-Cy5.5, anti-CD40-PE-Cy7, anti-BAFF-PE and anti-CCR4-Brilliant Violet^TM^ 510 (BioLegend, San Diego, CA). For the CD4+ T cell phenotype, the following antibodies were used: anti-CD4-VioBlue, anti-CD3-VioGreen, anti-CD45RA-FITC, anti-CD45RO-PerCP-Vio700, anti-IFN-γ-PE, anti-IL-4-PE, anti-IL-21-PE, anti-STAT3 pS727-PE (Miltenyi), anti-CCR5-PerCP-Cy5.5, anti-CXCR3-PE-Cy7, anti-CD40L- Brilliant Violet^TM^ 510, anti-CCR6-PerCP-Cy5.5, anti-CCR4-Brilliant Violet^TM^ 510, anti-CXCR5-PE-Cy7, anti-CCR7-FITC, anti-IL-10-APC, anti-Bcl-6-APC (BioLegend), anti-IL17A-APC and anti-ICOS-APC (eBioscience, San Diego, CA). For CFSE staining, 10^6^–10^7^ sorted CD4^+^CD45RA^+^ and CD4^+^CD45RO^+^ T cells were labeled for 10 min at room temperature with 0.5 μM CFSE (eBioscience) in 1 ml PBS. Cells were washed three times and resuspended in an X-vivo 15 free serum medium (Lonza). BD GolgiStop and BD Perm/Wash were used for intracellular staining as per the manufacturer’s instructions (BD Biosciences). All cells were analyzed using an LSRFortessa cell analyzer (BD Biosciences). The analysis of the data was conducted using FlowJo version 10 (FlowJo LLC, Ashland, OR). Cells were first gated based on FSC and SSC. Positive thresholds were set based on isotype controls.

### Elisa

MCP-1, IL-6, IFN-γ, IL-4 and IL-17A levels in cell culture supernatants were measured using multiplex ELISA (Ciraplex assay; Aushon Biosystems, Billerica, MA). Plates were read with a Cirascan^TM^ imager (Aushon Biosystems) and analyzed with Cirasoft^TM^ software (Aushon Biosystems). These results were confirmed by a single ELISA (BioLegend, San Diego, CA). IL-21 was measured using a single ELISA (eBioscience) according to the manufacturer’s instructions.

### Immunohistochemical and immunofluorescence staining of paraffin sections

Briefly, the tissue was deparaffinized in xylene and rehydrated in different concentrations of ethanol. After antigen retrieval using pressure cooking in 0.01 M citrate buffer at pH 6.0, endogenous peroxidase activity was blocked (EnVision FLEX system; Dako, Mississauga ON). For immunohistochemical staining, the slides were stained with mouse anti-CXCR5 (Abcam, Toronto, ON) overnight at 4 °C. Endogenous biotin was blocked (Biotin Blocking system; Dako), then slides were incubated successively with EnvisionFlex + mouse (linker) and EnvisionFlex/HRP (Dako). Antibody revelation was performed with 3,3′-Diaminobenzidine (DAB) chromogen and counterstain with Mayer’s Hematoxylin (Dako). For immunofluorescence, the sections were blocked and stained with mouse anti-CXCR5 (Abcam) and rabbit anti-CD3 (Abcam) overnight at 4 °C. After washing with phosphate-buffered saline, the sections were stained with secondary antibodies goat anti-rabbit Alexa Fluor 488-conjugated and goat anti-mouse Alexa Fluor 594-conjugated (Invitrogen) for 1 h at room temperature. Slides were mounted in a glycine-glycerol buffer. All sections were analyzed on a multicolor motorized fluorescence microscope IX83 (Olympus, Richmond Hill, ON) equipped with a dual color and monochrome CCD Olympus camera DP80 and images were analyzed using the Olympus cellSens Dimensions Software.

### Patients

Blood samples were obtained as part of a single-center, observational, cohort study with a prospective collection of biological samples and clinical and pathological data. Between January 2012 and October 2015, 55 participants were enrolled under the approved guidelines of the Ethics Committee of University Health Center (CHU) of Quebec (project 2002-1001, A12-06-1001/Renewal F9-25020). Written informed consent was obtained from each participant. Patients were invited to provide blood and urine samples at the time of a graft biopsy. PBMCs were isolated and stored for all patients. Biopsies were evaluated by one of two pathologists (J.R., E.L.) using the Banff classification of renal allograft pathology^42,43^. No patients were lost to follow-up. The reported clinical and research activities are consistent with the Principles of the Declaration of Istanbul.

### Statistical Analysis

All data were analyzed using the Mann-Whitney test or Wilcoxon signed-rank test. All analyses were two-tailed, and a P-value less than 0.05 was considered statistically significant.

### Data Availability

The datasets generated during and/or analyzed during the current study are available from the corresponding author on reasonable request.

## Electronic supplementary material

Supplementary Figures
