# Supplementary material for: Allogeneic dendritic cells stimulated with antibodies against HLA class II polarize naive T cells in a follicular helper phenotype
Source: Sci Rep. 2018 Mar 5;8:4025. doi: 10.1038/s41598-018-22391-w (PMC5838222; doi:10.1038/s41598-018-22391-w)
Supplement: Supplementary file 1 — Supplementary Figures [file 41598_2018_22391_MOESM1_ESM.pdf]

Allogeneic Dendritic Cells Stimulated with Antibodies Against HLA Class II Polarize Naive T Cells in a Follicular Helper Phenotype

Olivier Désy PhD[1], Stéphanie Béland MSc[1], Patrice Vallin MSc[1], Julie Riopel MD FRCPC[2], Eva Latulippe MD FRCPC[2], Eric Wagner PhD[3] and Sacha A. De Serres MD SM FRCPC[1]*

Supplementary Figure S1.

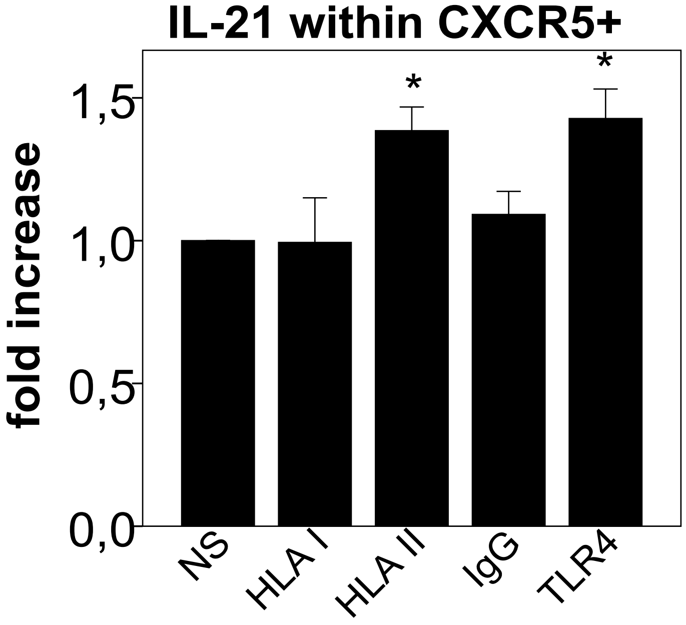

Supplementary Figure S1. HLA-II-moDCs polarize $CD4^+$ naive T cells into a Tfh phenotype. In all experiments, $CD4^+$ naive T cells were cocultured with stimulated moDCs for 6 days. Summary analysis of intracellular IL-21 expression (gated on $CD3^+CD4^+CXCR5^+$cells). Values are expressed as the fold increase compared with levels in untreated cells (mean ± SEM, n=9). *, p<0.05; NS, non-stimulated; IgG, isotype control.

Supplementary Figure S2.

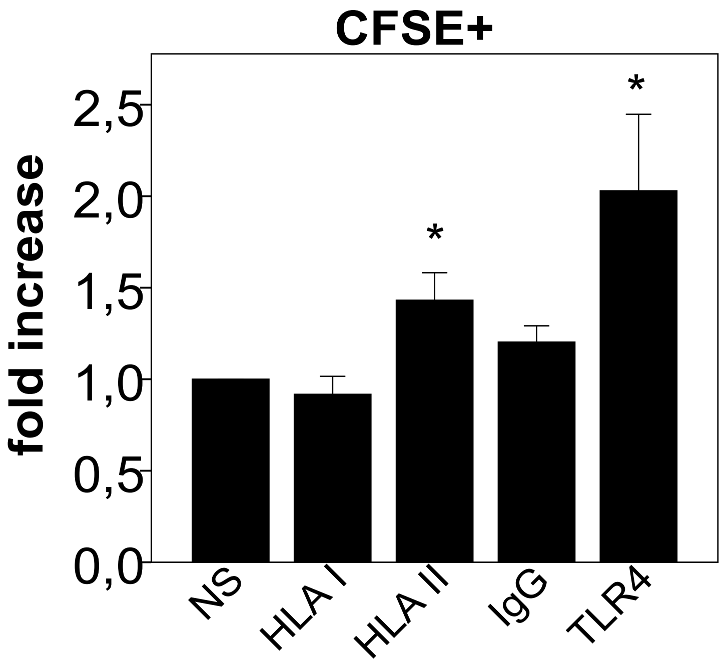

Supplementary Figure S2. HLA-II-moDCs increase T cell proliferation in coculture. In all experiments, $CD4^+$ naive T cells were cocultured with stimulated moDCs for 6 days. Summary analysis of the CFSE positivity in the $CD4^+$ T cells. Values are expressed as fold increase compared with unstimulated cells (mean ± SEM, n=7). *, $p<0.05$; NS, non-stimulated; IgG, isotype control.

Supplementary Figure S3.

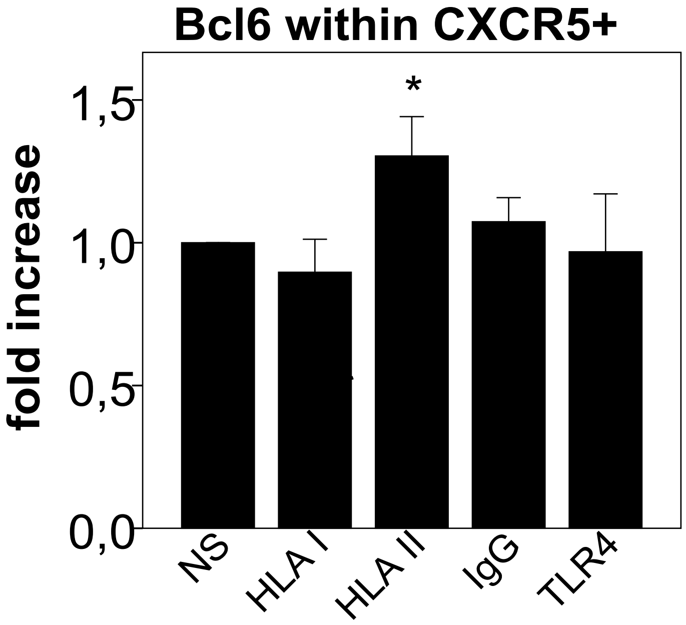

Supplementary Figure S3. HLA-II-moDCs polarize CD4$^+$ naive T cells into a Tfh phenotype. In all experiments, CD4$^+$ naive T cells were cocultured with stimulated moDCs for 6 days. Summary analysis of intracellular Bcl6 expression (gated on CD3$^+$CD4$^+$CXCR5$^+$cells). Values are expressed as the fold increase compared with levels in untreated cells (mean ± SEM, n=9). *, $p<0.05$; NS, non-stimulated; IgG, isotype control.

Supplementary Figure S4.

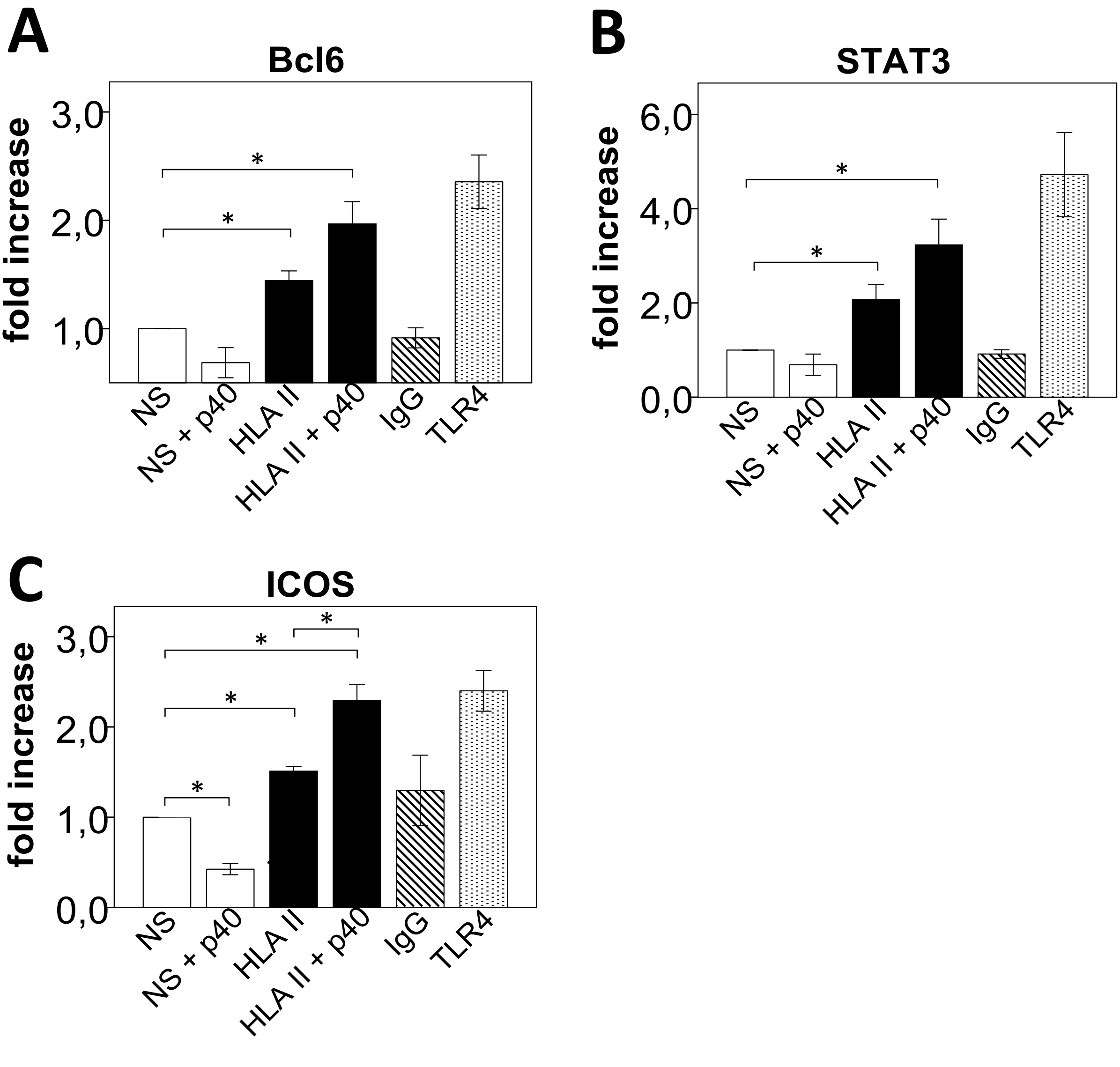

Supplementary Figure S4. Effect of p40 on the expression of Tfh-specific transcription factors and surface phenotype. Flow cytometry analysis of (A) Bcl6, (B) STAT3 and (C) ICOS in $CD4^+$ T cells stimulated with allogeneic unstimulated moDCs (NS), NS + p40 subunit of IL-12, HLA-II-moDCs (HLA II), HLA II + p40 subunit of IL-12, IgG2a-moDCs (IgG) and TLR4 for 6 days (mean ± SEM, n=5). *, $p<0.05$; NS, non-stimulated; IgG, isotype control

Supplementary Figure S5.

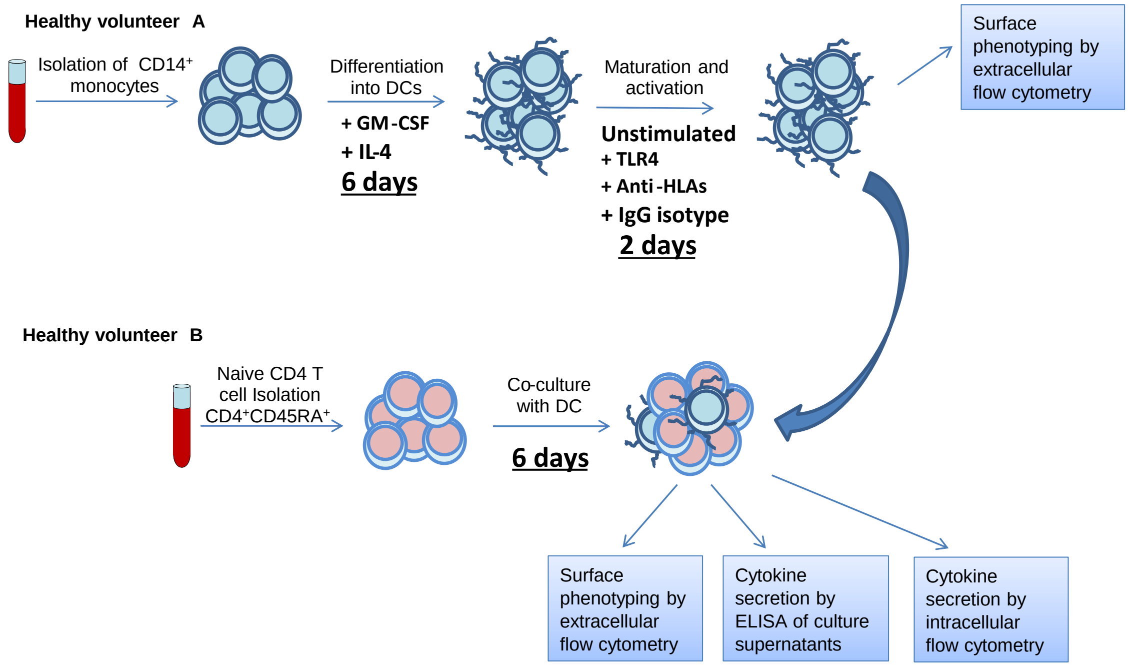

Supplementary Figure S5. ***In vitro*** **model used for the generation of Tfh cells**
